# Immunoregulation by Mesenchymal Stem Cells: Biological Aspects and Clinical Applications

**DOI:** 10.1155/2015/394917

**Published:** 2015-04-19

**Authors:** Marta E. Castro-Manrreza, Juan J. Montesinos

**Affiliations:** Mesenchymal Stem Cells Laboratory, Oncology Research Unit, Oncology Hospital, National Medical Center, IMSS, Avenue Cuauhtémoc 330 Col. Doctores, 06720 Mexico City, DF, Mexico

## Abstract

Mesenchymal stem cells (MSCs) are multipotent cells capable of differentiation into mesenchymal lineages and that can be isolated from various tissues and easily cultivated *in vitro*. Currently, MSCs are of considerable interest because of the biological characteristics that confer high potential applicability in the clinical treatment of many diseases. Specifically, because of their high immunoregulatory capacity, MSCs are used as tools in cellular therapies for clinical protocols involving immune system alterations. In this review, we discuss the current knowledge about the capacity of MSCs for the immunoregulation of immunocompetent cells and emphasize the effects of MSCs on T cells, principal effectors of the immune response, and the immunosuppressive effects mediated by the secretion of soluble factors and membrane molecules. We also describe the mechanisms of MSC immunoregulatory modulation and the participation of MSCs as immune response regulators in several autoimmune diseases, and we emphasize the clinical application in graft versus host disease (GVHD).

## 1. Introduction

Mesenchymal stem cells (MSCs), also referred to as multipotent mesenchymal stromal cells, have been a focus of recent research, partially because they are an extraordinary model for investigating the biological mechanisms that allow a cellular population to generate diverse cell types and because they are a potential tool in cellular therapies for several clinical applications. MSCs can differentiate into mesenchymal lineages and secrete cytokines and growth factors with paracrine effects that favor the regeneration of damaged tissues [1, 2]. Several studies have demonstrated that MSCs possess an immunoregulatory function* in vitro* and* in vivo* and that this property suggests clinical applications in the regulation of immunocompetent cell responses [2, 3]. This review addresses current knowledge of the biological aspects involved in MSC immunoregulatory capacity and the clinical focus of these characteristics that allows these cells to be used in the treatment of several diseases with an immune component involved. This review culminates with a clinical description of the diseases treated with MSCs as a component of cell therapy procedures.

## 2. Definition and Characteristics of MSCs

MSCs are adult stem cells that are initially isolated from bone marrow (BM) [4] and can generate stromal BM components, such as adipocytes, reticular cells, and osteoblasts, whereas in conjunction with additional cellular components, MSCs maintain hematopoiesis [5]. MSCs proliferate* in vitro* as adherent, colony-forming cells with a high capacity for self-renewal and proliferation [4, 5]. Because there is no definite marker of MSCs, the International Society for Cellular Therapy has established minimum criteria that these* in vitro* cell populations must fulfill and certain characteristics to be considered MSCs. The cells must be positive for CD105, CD73, and CD90, express low levels of MHC-I, and be negative for MHC-II, CD11b, CD14, CD34, CD45, and CD31. Additionally, these cells must be capable of differentiation into osteoblasts, adipocytes, and chondroblasts* in vitro* [5, 6].

MSCs have been isolated from multiple tissues: skeletal muscle, adipose tissue (AT), synovial membranes, dental pulp, periodontal ligaments, cervical tissue, menstrual blood, Wharton's jelly (WJ), umbilical cord (UC), umbilical cord blood (UCB), amniotic fluid, placenta (PL), and fetal tissues such as blood, liver, and BM [7–10]. In most cases, isolated MSCs are heterogeneous in proliferation and differentiation, although all express the characteristic MSC marker profile. MSCs cultivated* in vitro* possess three biological properties that qualify them for use in cellular therapy: (a) broad potential of differentiation, (b) secretion of trophic factors that favor tissue remodeling, and (c) immunoregulatory properties [2]. These traits make MSCs potential tools in many conditions. Furthermore, MSCs differentiate into different mesodermal lineages (adipocytes, chondrocytes, osteocytes, fibroblasts, and myocytes) [5]. Because of this potential for differentiation, MSCs were initially used in the treatment of imperfect osteogenesis [11] and myocardial damage [12]. The benefits observed in these initial cell therapy protocols were thought to be the result of osteogenic and myogenic differentiation [3]. The current understanding is that, in addition to diverse mesodermal differentiation capacity, MSC benefits arise primarily from the secretion of trophic factors and immunoregulatory capacity [1–3].

## 3. Immunoregulatory Properties of MSCs

Multiple studies have demonstrated the immunoregulatory properties of MSCs. MSCs profoundly affect immune response through their interactions with the cellular components of the innate (natural killer cells (NK)) and adaptive (dendritic cells (DCs), B lymphocytes, and T lymphocytes) immune system. MSC immunoregulation can occur through cellular contact and/or the secretion of diverse factors [13–17]. Because of these properties, MSCs can prevent the inappropriate activation of T lymphocytes and generate a tolerogenic environment during wound repair or stop an immune response during healing, thus contributing to the maintenance of immune homeostasis [2, 3]. Below, we describe the immunoregulatory effects of MSCs on specific immune cells with special emphasis on the effect of MSCs on T lymphocytes because of their role as effector cells in many diseases with an immune component.

### 3.1. Immunosuppressive Effects on Immunocompetent Cells

#### 3.1.1. T Lymphocytes

When lymphocytes are activated, they proliferate and differentiate to fulfill their effector functions. MSCs modulate each of these phases, thus influencing T lymphocyte immune response. The phases in which T cells are vulnerable to MSC immunoregulation, recognizing from a biological perspective that there are no obvious limits between phases, are described below. 

*(1) Activation.* During activation, T lymphocytes express and secrete molecules characteristic of this phase, such as CD25, CD69, CD38, cytotoxic T lymphocyte antigen-4 (CTLA-4), and human leukocyte antigen-DR (HLA-DR) and in addition the cytokines Interferon-*γ* (IFN*γ*), tumor necrosis factor (TNF*α*), and IL-2, among others [18]. Currently, there are contradictory results regarding the effect of MSCs on T lymphocyte activation. Some studies have observed that BM-MSCs prevent the expression of the early activation markers CD25 and CD69 in T cells stimulated with phytohemagglutinin (PHA) [19, 20], whereas other studies describe no effect by BM-MSCs on the expression of these molecules [16, 21]. Such contradictory results may be because of differences in the population of T lymphocytes studied. With this understanding, the activation of peripheral blood mononuclear cells (PBMC) with PHA in the presence of BM-MSCs results in lower numbers of CD4^+^ and CD8^+^ that express CD25, CD38, and CD69 [20]. Employing the identical model, other authors have described a smaller proportion of cells expressing CD3^+^CD25^+^ and CD3^+^CD38^+^ [19]. Contradictory results report that the activation of PBMC with antibodies in the presence of BM-MSCs does not modify the expression of CD25 or CD69 in CD4^+^ and CD8^+^ populations [21]. Similarly, no change was reported in the expression of CD25, CD69, or CTLA-4 in studies of populations enriched with CD4^+^ and CD8^+^ lymphocytes activated by alloantigens [16]. However, a recent study using populations enriched with CD3^+^ T lymphocytes activated with anti-CD2/CD3/CD28 in the presence of BM-MSCs showed an increase in the expression of CD69^+^ in CD4^+^ and CD8^+^ T lymphocyte populations [22]. These results emphasize the importance of the cellular context in MSC immunoregulation.

The effects of MSCs on the secretion of cytokines by activated T lymphocytes are also described by contradictory results. Some studies have demonstrated that the presence of MSCs diminishes [14, 21] or increases [23, 24] significantly the secretion of IFN*γ* by activated T lymphocytes. Nonetheless, it has been described that the effects of MSCs on IFN*γ* secretion depend on the source of the lymphocyte population studied [23]. In this study, the authors demonstrated that the activation of CD3^+^ T lymphocytes with anti-CD3/CD28 in the presence of MSCs from adipose tissue resulted in an increase in IFN*γ*, which was an effect that was not observed when PBMC were activated with the identical stimulus. Our laboratory recently demonstrated that the activation of CD3^+^ T cells with antibodies in the presence of BM-MSCs, UCB-MSCs, and PL-MSCs also resulted in an increase in IFN*γ* in cocultures [24]. This observed effect might be related to the generation of IFN*γ*-producing T regulatory cells (Tregs) [25]. 

**Figure 1:**
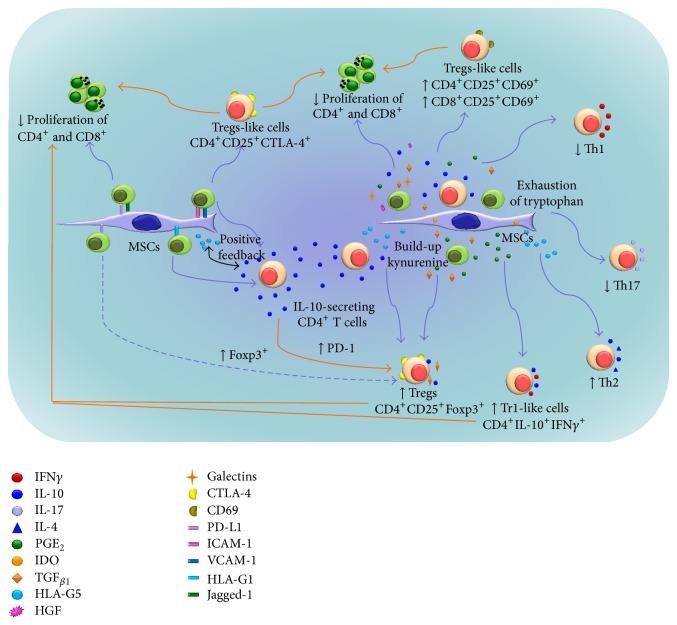
Immunoregulatory effect of MSCs on T lymphocytes. MSCs can induce the sustained expression of CTLA-4 and CD69 activation molecules on T cells, which have been related with generation of cells with immunoregulatory properties. Cell-to-cell contact seems to be required for the increase of CTLA-4 expression. Dependent and independent mechanisms of cellular contact are involved in the decrease of proliferation of CD4^+^ and CD8^+^ T cells and in generation of Foxp3^+^ Tregs by MSCs. Cytokines such as IL-10 can stimulate the expression and secretion of HLA-G5 by MSCs and in turn it stimulates the secretion of IL-10 in a positive feedback loop. The initial contact between MSCs and T lymphocytes seems to be required for initiation of the feedback loop. PGE_2_ secreted by MSCs is involved in generation of Tr1 cells. HLA-G5 supports differentiation of Th2 cells and IDO decrease differentiation of Th17.

*(2) Proliferation.* The effect of MSCs on the proliferation of T lymphocytes is independent of the activation method. The first studies to analyze the effects of BM-MSCs on the proliferation of T lymphocytes used irradiated MSCs that were cocultivated with alloantigen-stimulated PBMC. These studies reported that MSCs inhibited T-cell proliferation in a dose-dependent fashion [13, 16, 17, 19]. However, in addition to inhibiting alloantigen-induced proliferation, MSCs can inhibit proliferation induced by polyclonal activators such as PHA [14, 26] or anti-CD3/CD28 [21, 23, 24]. The immunosuppressive effects of MSCs have been analyzed using total populations of PBMC and populations enriched in CD3^+^, CD4^+^, or CD8^+^ lymphocytes. Every case has demonstrated the capacity of MSCs to diminish proliferation [13, 16, 17, 19, 21, 23, 24]. Immunoregulation has been shown to be independent of the induction of apoptosis [16, 27] and is performed through mechanisms dependent and independent of cellular contact. Among the secreted factors identified are transforming growth factor beta 1 (TGF_*β*1_), hepatocyte growth factor (HGF), indoleamine-2,3-dioxygenase (IDO), prostaglandin E2 (PGE_2_), IL-10, and HLA-G5 [13, 17, 28], whereas programmed death-ligand 1 (PD-L1) and HLA-G1 are involved in contact-dependent mechanisms (Figure 1) [28–30]. Whether direct contact between MSCs and T lymphocytes is necessary for the inhibition of T-cell proliferation remains controversial. Some authors have suggested that MSCs act via an immunosuppressive mechanism independent of cell-to-cell contact [31, 32], whereas others have indicated that contact is required for efficient immunoregulation [24, 28–30, 33, 34]. However, the mechanism of MSC immunoregulation appears to depend on cellular populations, mode of activation, and the presence or absence of cell-to-cell contact [23, 32]. 

*(3) Differentiation and Effector Function*. Upon activation by the presence of pathogens or signs of damage, helper T cells CD4^+^ (Th0) differentiate into one of the following subtypes, depending on the T-cell microenvironment: Th1, Th2, Th17, or Tregs. Each population is characterized by the secretion of a set of cytokines whose function is essential to eliminate pathogens within the organism, resolve inflammation, and maintain immune homeostasis. Several studies have suggested that MSCs modulate the differentiation, function, and balance of these subpopulations and foster the development of an anti-inflammatory immune response (Figure 1) [14, 28, 35, 36]. The activation of naïve T cells (CD45RA^+^) in favorable conditions for the induction of Th1 or Th2 and in the presence of MSCs results in the inhibition of IFN*γ* secretion by Th1 cells and the increase of IL-4 secretion by Th2 cells [14]. Furthermore, MSCs inhibit the production of proinflammatory cytokines IL-17, IL-22, IFN*γ*, and TNF*α* and the differentiation of naïve CD4^+^ lymphocytes to Th17. Additionally, MSCs promote the secretion of IL-10 and the expression of the Foxp3 transcription factor, thus suggesting differentiation toward Tregs (Figure 1) [35]. Similarly, in a murine model, the presence of MSCs in the Th1 and Th17 differentiation processes favors differentiation to CD4^+^CD25^+^Foxp3^+^ Tregs. This effect was not observed when MSCs were added to cultures of mature Th1 or Th17 populations [36]. These results indicate that MSCs affect the differentiation and function of inflammatory T lymphocyte populations in their capacity to produce proinflammatory cytokines and also in the induction of a Tregs phenotype.

Several studies have described the role of MSCs in the induction of distinct Tregs populations [14, 28, 37–40]. In an initial study analyzing the participation of BM-MSCs in the differentiation of Tregs populations, Maccario et al. observed that the presence of allogeneic and autologous MSCs, with respect to the responder T lymphocyte population in mixed lymphocyte culture (CML), induced a significant increase in the CD4^+^CD25^bright^ T lymphocyte population. However, only allogeneic MSCs favored an increase in CD4^+^CD25^+^CTLA-4^+^ populations [37]. In the same year, a different study observed that PBMC activation by IL-2 in the presence of MSCs increased the proportion of CD4^+^CD25^+^ T lymphocytes [14]. Subsequently, Prevosto et al. showed that PBMC in coculture with BM-MSCs generated a population of cells that could inhibit T lymphocyte proliferation induced by alloantigens or polyclonal activators (anti-CD3 or PHA), and this effect required cell-to-cell contact. These Tregs populations could also be derived from CD4^+^ or CD8^+^ cells. The authors observed that the increase in Foxp3 mRNA expression occurred only in the Tregs populations derived from CD4^+^ lymphocytes [38]. MSCs can induce and maintain the function and phenotype of Tregs derived from CD3^+^, CD3^+^CD45RO^+^, or CD3^+^CD45RA^+^ T lymphocyte populations. Beginning with a CD3^+^ population, BM-MSCs primarily recruit Tregs from virgin CD3^+^CD45RA^+^ lymphocytes. The authors also demonstrated that the presence of BM-MSCs maintained Foxp3 expression in Tregs [40]. MSCs from UC also have capacity to induce generation of Tregs [34].

Furthermore, MSCs can induce regulatory T type 1- (Tr1-) like cells characterized by IFN*γ* and IL-10 secretion. Through an* in vivo* transplant-induced arteriosclerosis model (obstructed arteries), Jui et al. demonstrated that the local administration of BM-MSCs could prevent this pathology through a local increase in IFN*γ* and IL-10 [41]. In a subsequent* in vitro* study, the identical laboratory demonstrated that BM-MSCs favored the generation of Tr1 lymphocytes with an IL-10^+^IFN*γ* ^+^CD4^+^ phenotype mediated by PGE_2_ and IDO (Figure 1) [25]. MSC participation in T lymphocyte subpopulation equilibrium has also been observed in human* in vivo* studies. Patients who have received MSCs for the treatment of GVHD show subsequent increases of CD4^+^CD25^+^Foxp3^+^ and Tr1 populations and decreases of Th17^+^ [42]. Similarly, the administration of MSCs to patients with systemic lupus erythematous induced an increase in CD4^+^CD25^+^Foxp3^+^ Tregs in the peripheral blood [43]. Increases in this Tregs population have also been observed in kidney transplant patients, which were transplanted with autologous MSCs [44].

#### 3.1.2. Dendritic Cells

DCs are the most important antigen presenting cells in the body. These cells are derived from BM-CD34^+^ cells* in vivo* and from monocytes stimulated with IL-4 and granulocyte macrophage colony-stimulating factor (GM-CSF)* in vitro*. The primary function of DCs is to process and present antigens to virgin and memory T cells, although they also interact with other immune components such as B lymphocytes and NK cells. The individual DCs must mature to initiate an appropriate immune response, and during the maturation process, DCs increase the membrane expression of MHC-II and T-cell costimulatory molecules CD80 and CD86. Immature DCs can not only activate T cells but also induce tolerance [45].

**Figure 2:**
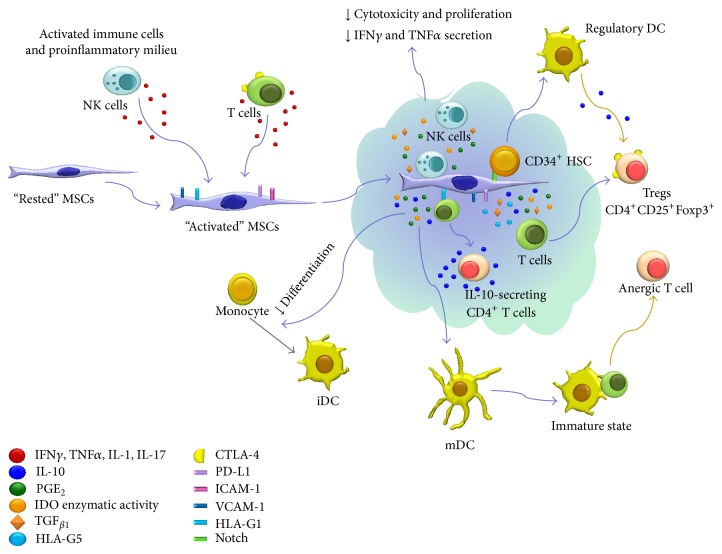
Immunoregulatory effects of MSCs on immune cells. Proinflammatory cytokines such as IFN*γ* secreted by activated NK cells and T lymphocytes support MSCs-mediated immunoregulation and can increase or induce the production of immunosuppressive molecules. IDO, PGE_2_, TGF_*β*1_, and membrane molecules are mainly involved in MSCs immunoregulation on NK cells. IDO, PGE_2_, and IL-10 are involved in the decrease of differentiation of monocytes into iDCs and may push mDCs toward an immature state, which results in T-cell anergy and inappropriate activation of T lymphocytes. MSCs induce the differentiation of CD34^+^ hematopoietic progenitors into a population of regulatory DCs, which in turn stimulate the generation of Foxp3^+^ Tregs.

MSCs can affect the recruitment, maturation, and function of DCs. MSCs can significantly reduce monocyte differentiation into DCs, affecting the upregulation of CD1a, CD40, CD80, CD86, and HLA-DR (Figure 2) [15, 37, 46–48]. This reduction is performed through the secretion of factors [15, 46] and is a reversible process because these monocytes then differentiate normally at the removal of MSCs [15]. When immature DCs (iDCs) derived from monocytes-MSC cocultures were activated with lipopolysaccharide (LPS) to induce their final differentiation, they expressed lower levels of the maturation marker CD83 and costimulatory molecules CD80 and CD86. These results suggest that MSCs can maintain DCs in an immature state [15]. However, Spaggiari et al. showed that MSCs do not affect direct LPS-induced maturation of DCs in cocultures, because there was no change in CD80, CD83, and CD86 expression [47]. These results suggest that MSCs exert a strong inhibitory effect on the differentiation process from monocytes to iDCs but not on the LPS-induced maturation of iDCs to mature DCs (mDCs). In addition, mDCs cocultured with MSCs show a diminished expression of HLA-DR, CD1a, CD80, and CD86, thus suggesting that MSCs may push mDCs toward an immature state with a reduced stimulatory capacity (Figure 2) [47].

Additionally, MSCs affect the secretion of several cytokines that are key to DCs maturation. Aggarwal and Pittenger observed that MSCs inhibit the secretion of TNF*α* by DCs activated by LPS. The inhibition of TNF*α* secretion by DCs inhibits their maturation, migration to the lymph nodes, and their capacity to stimulate alloreactive T lymphocytes, because of the alteration in the expression of several receptors that are necessary to capture and process antigens [14]. MSCs also inhibit the DCs secretion of IL-12 [15, 47, 48]. The insufficient production of IL-12 is associated with the induction of T cell anergy and tolerance [15, 26, 47]. Human BM-MSCs that act through Notch can induce the differentiation of CD34^+^ hematopoietic progenitors into a population of regulatory DCs with specific properties: (1) the expression of high levels of IL-10 mRNA and low expression of IL-2 mRNA; (2) the capacity to inhibit alloreactive T-cell proliferation and function; and (3) the capacity to induce Tregs differentiation characterized by expression of Foxp3 and TGF_*β*1_ mRNA (Figure 2) [49].

#### 3.1.3. NK Cells

NK cells are important in innate immunity and participate in the body's defenses against infections and cancer. NK cells perform their effector function through the secretion of cytokines, such as IFN*γ*, TNF_*β*_, and GM-CSF, and possess cytotoxic activity both spontaneous and antibody-dependent [50]. NK function is regulated by the equilibrium of signals transmitted by activator and inhibitor receptors that interact with specific HLA molecules on target cells. Thus, HLA-class I negative or HLA-class I-mismatched cells represent potential targets of NK cells [27, 50]. MSCs affect the phenotype, proliferation, cytotoxic potential, and cytokine secretion of NK cells (Figure 2). When activated by IL-2, NK cells secrete IFN*γ*, but when activated in the presence of MSCs, IFN*γ* secretion significantly decreases [14]. Furthermore, NK cells activated by IL-2 and alloantigens in the presence of MSCs show diminished proliferation and lytic activity [16, 51]. IL-15 is another cytokine that promotes the proliferation, survival, and effector function of NK cells, but through factor secretion, MSCs can inhibit IL-15 induced proliferation [27]. However, MSCs and NK cell contact are necessary to inhibit NK cytotoxicity in tumor cell lineages [27].

#### 3.1.4. B Lymphocytes

B lymphocytes are involved in the adaptive immune response. These cells are responsible for humoral immunity and are specialized for antibody production [52]. Few studies have analyzed the effects of MSCs on B lymphocytes; however, MSCs diminish B-cell proliferation by cell cycle arrest in the G0/G1 phase and not by inducing apoptosis [53]. A recent study demonstrated that effect of MSC on B lymphocytes proliferation with CpG is not direct and requires presence of CD3^+^ T cells [54]. MSCs can also affect B-cell differentiation because IgM, IgG, and IgA production are diminished [53–56]. Furthermore, MSCs modify the chemotactic properties of B lymphocytes, because expression changes in their chemokine receptors including CXCR4, CXCR5, and CCR7 were induced by MSCs [53].

### 3.2. Soluble Factors Involved in MSC Immunoregulation

#### 3.2.1. TGF_*β*1_ and HGF

The first molecules described in the MSC-mediated immunoregulation of alloantigen-activated T lymphocytes were TGF_*β*1_ and HGF. Both cytokines can independently diminish alloantigen-activated T lymphocyte proliferation, although proliferation can be partially reestablished through blocking with antibodies [20, 31]. MSCs constitutively express TGF_*β*1_ and HGF, which appear to act synergistically [17]. TGF_*β*1_ is involved in the MSC-mediated generation of CD4^+^CD25^+^Foxp3^+^ Tregs (Figure 1) [39] and in the decreased proliferation of NK cells [27]. These results suggest that TGF_*β*1_ and HGF, in addition to other mechanisms, participate in the suppression of MSC-mediated proliferation in mixed lymphocyte culture (MLC).

#### 3.2.2. Indoleamine-2,3-dioxygenase

IDO is an enzyme that catalyzes the conversion of the amino acid tryptophan to kynurenine. The inhibition of T lymphocyte proliferation is because of the exhaustion of tryptophan or the build-up of kynurenine [57]. There is experimental evidence supporting both hypotheses. Thus, the addition of exogenous tryptophan has been shown to reestablish alloantigen-activated T-cell proliferation in the presence of MSCs [13]. Similarly, the addition of kynurenine to MLC inhibits proliferation without MSCs, however such inhibition is lower when compared with that observed in cultures in the presence of MSCs [17]. The use of competitive inhibitors of IDO reduces MSC immunosuppressive effects on alloantigen-activated CD4^+^ T lymphocytes [16]. Notably, the reestablished proliferation does not reach the levels observed in MLC without MSCs, thus suggesting the presence and participation of additional mechanisms [13]. The exhaustion of tryptophan by IDO participates in the inhibition of Th17 differentiation; however, this mechanism and the build-up of kynurenine are involved in the IDO generation of Foxp3^+^ Tregs [57]. Furthermore, IDO is involved in the decrease of proliferation and cytotoxic activity of NK cells activated by IL-2 in the presence of MSCs [51] and also in the inhibition of maturation and functional activity of DCs (Figure 2) [45].

#### 3.2.3. Prostaglandin E_2_

PGE_2_ also plays a role in MSC-mediated immunoregulation. PGE_2_ is a lipid mediator derived from the conversion of arachidonic acid to prostaglandin through COX1 and COX2 enzyme action [58]. These enzymes, with PGE_2_, are constitutively expressed by MSCs, although their expression increases in an inflammatory environment [14, 17]. PGE_2_ has been shown to diminish proliferation, stimulate the secretion of IL-4 and IL-10, and promote CD4^+^CD25^+^Foxp3^+^ and IL-10^+^IFN*γ* ^+^CD4^+^ Tregs differentiation (Figure 1) [25, 59]. Several studies have shown that PGE_2_ is a MSC effector molecule; synthesis can be blocked with indomethacin or NS-398, and activated T-cell proliferation increases, but not similar to levels observed of T-cell proliferation in absence of MSCs [14, 17, 32, 60]. PGE_2_ is involved in the decrease of differentiation of monocytes into DCs [47] and the decrease of proliferation and cytotoxic activity of NK cells activated by IL-2 in the presence of MSCs (Figure 2) [51].

#### 3.2.4. IL-10

It has been reported that IL-10 is expressed by human [17] and murine [61] MSCs and that TLR3 ligand increases the IL-10 secretion by human MSCs [62]. However, other authors have showed that murine [16, 63] and human MSCs do not express IL-10 [64]. Despite these conflicting results, some studies have reported a high concentration of IL-10 in the supernatant from cocultures of fetal or adult MSCs and immune cells and their participation in the immunosuppression by MSCs in such cocultures have been well demonstrated [38]. In that regard, some authors have showed that cell-to-cell contact between BM-MSCs and T cells appears vital in the concentration increase of IL-10 in the supernatant [24, 38, 64]. Participation of IL-10 in BM-MSC-mediated immunoregulation and in Tregs generation has been demonstrated through the use of antibodies [17, 20]. IL-10 downregulates Th1 cytokine expression and can stimulate the expression and secretion of HLA-G5, which is another important molecule in MCS-mediated immunoregulation [28]. A recent study reported an increase in immunosuppressive capacity of CD4^+^CD25^+^ Tregs cocultured with BM-MSCs, which is due to a high expression of PD-1 on Tregs stimulated by IL-10 present in the coculture supernatant (Figure 1) [65]. Furthermore, IL-10 is also involved in the decrease of maturation and function of DCs, inhibiting the ability of DCs to produce IL-12 (Figure 2) [61].

#### 3.2.5. HLA-G5

HLA-G molecules are nonclassic HLA molecules characterized by a limited allelic polymorphism and a tissue-specific expression pattern. There are membrane-bound isoforms (HLA-G1, G2, G3, and G4) and soluble isoforms (HLA-G5, G6, and G7) [66]. BM-MSCs express the membrane-bound isoform HLA-G1 and the soluble isoform HLA-G5 [28, 30]; expression of both molecules is promoted by IL-10 [28]. Similarly, HLA-G5 stimulates the secretion of IL-10 in a positive feedback loop. The simultaneous use of antibodies against both molecules nearly reestablishes proliferation of alloantigen-activated PBMC in the presence of MSCs [28, 67]. Direct contact between MSCs and T cells is required to establish the positive feedback loop and subsequent generation of an immunosuppressive environment, which is further exacerbated by the generation of the CD4^+^CD25^+^Foxp3^+^ Tregs induced by both molecules (Figure 1) [28].

#### 3.2.6. Galectins

Galectins are a family of proteins that bind specifically to *β*-galactoside. Eleven of the 15 galectins are distributed in lymphoid and nonlymphoid tissues. Galectins participate in the regulation of cellular homeostasis in both adaptive and innate immunity as immunostimulators or immunosuppressors [68]. Several galectins have been implicated in MSC-mediated immunoregulation as is described below. Galectin-1 mRNA and protein are constitutively expressed and released into culture medium by MSCs [69]. The silencing of Galectin-1 expression using siRNA [70] or antibodies [71] reestablishes proliferation of PBMC activated by mitogens or alloantigen, thus indicating that Galectin-1 participates in MSC immunoregulation. Similarly, MSCs express Galectin-3, a molecule known to regulate T-cell proliferation, adhesion, and migration [68]. The inhibition of Galectin-3 expression in MSCs with siRNA reduces immunosuppressive capacity on alloantigen-activated T lymphocytes [72]. Ungerer et al. recently demonstrated that Galectin-9 also participates in MSC-mediated immunoregulation. The authors observed that MSCs express higher levels of Galectin-9 in an inflammatory environment, and through the use of antibodies, the authors described the participation of Galectin-9 in the MSC-mediated proliferation reduction of stimulated T and B lymphocytes [73, 74].

### 3.3. Membrane Molecules Involved in MSC-Mediated Immunoregulation

The following membrane molecules participate in MSC-mediated immunoregulation: the PD-1/PD-L1 pathway, HLA-G1, Jagged-1, and adhesion molecules, such as intercellular adhesion molecule 1 (ICMA-I) and vascular cell adhesion molecule 1 (VCAM-I) (Figure 1). PD-L1 (B7-H1/CD274) and its receptor (PD-1/CD279) are components of a T lymphocyte costimulatory pathway that releases inhibitory and regulatory signals upon activation. The pathway is involved in tolerogenesis and immune response termination to avoid tissue damage [75]. The PD-1/PD-L1 pathway is activated in the event of a persistent antigenic stimulus, as occurs with self-antigens, chronic viral infection, or tumors. The activation of this pathway prevents autoimmunity and directly contributes to the immunosuppressive microenvironment observed in tumors through T-lymphocyte regulation [75]. In murine models [29] and human MSC from PL [76], UC [34], and BM [34, 65, 77], it has been observed that IFN*γ* induce an increase of PD-L1 expression and has been demonstrated the participation of PD-L1 in MSC-mediated immunosuppression of T cell proliferation through the use of monoclonal antibodies [29, 75, 76]. Future studies will be necessary to determine the participation of PD-L1 expressed on MSCs in the expression of Foxp-3 in T cells. Interestingly, an increase in the expression of PD-L1 receptor (PD-1) has been observed in activated CD4^+^CD25^+^ Tregs cocultured with BM-MSCs, which is associated with a high immunosuppressive capacity. The same increase in PD-1 was observed in CD4^+^CD25^−^ T cells, but in contrast with Tregs, it is associated with apoptosis [65, 77].

HLA-G1 is similarly important in MSC-mediated immunoregulation. Giuliani et al. [30] demonstrated that the reduced proliferation in anti-CD3/CD28-activated T lymphocytes in the presence of MSCs derived from BM or fetal liver was primarily driven by cell-to-cell contact and that MSCs expressed higher levels of HLA-G1 in coculture. Furthermore, the use of antibodies specific to this molecule nearly reestablished T lymphocyte proliferation and reduced concentration of IL-10 in cocultures, which shows the relevance of cell-to-cell contact [30]. Similar studies have reported the importance of cell-to-cell contact between MSCs and T cells in such mechanisms [24, 38, 64]. It appears that cell-to-cell contact in cocultures promotes expansion of CD4^+^ T cells which produce IL-10 and increase the expression of CD210 (IL-10 receptor, subunit A) on CD4^+^ T cells but not on CD8^+^ T cells and MSCs [64]. These results suggest that through cell-to-cell contact between MSCs and T cells a population of T cells whose secreted products contribute to the immunoregulatory environment generated by MSCs is formed (Figure 1).

The participation of the adhesion molecules ICAM-I and VCAM-I has been demonstrated using antibodies against both adhesion molecules, which are expressed by MSCs during T-cell immunoregulation. When ICAM-I and VCAM-I were blocked in a mouse model, anti-CD3-activated splenocyte proliferation was partially reestablished [78]. Additionally, the expression of ICAM-I and VCAM-I increased when MSCs were exposed to IFN*γ* [35]. These results suggest that MSCs increase the capacity to recruit inflammatory T-lymphocyte populations and modulate its own function toward a regulatory phenotype as was demonstrated in the Th17 population [35]. Furthermore, adhesion molecules could be involved in T-cells immunoregulation through induction of CTLA-4 expression on T cells, which has been previously demonstrated [79, 80]. Our laboratory recently demonstrated that cell-to-cell contact between activated CD3^+^ lymphocytes and MSCs from BM or UCB increased the expression of CTLA-4 (Figure 1) [24].

Through different mechanisms CTLA-4 is a negative regulator of immune response. It has been demonstrated that interaction between CTLA-4 with CD80/B7-1 and CD86/B7-2 expressed in DC induces IDO upregulation by DC [57]. It is likely that a similar mechanism is present in CD4^+^CTLA-4^high^ T cells generated in cocultures with MSC [24, 37, 38]. This idea is supported by several evidences; activated T cells express CD80 and CD86, in particular the presence of MSC induces increased expression of CD86 on CD4^+^ T cells [81], and it has been shown that the interaction of these molecules with CTLA-4 induced IDO expression in CD4^+^ T cells [82].

The cell-to-cell contact between MSCs and CD3^+^ T cells is important in the immunosuppresion of B cells by MSCs [54]. This evidence suggest that inhibition of B-cell proliferation observed in cocultures with MSC is dependent of soluble factors produced during cell-to-cell contact between T cells and MSCs. Another molecule involved in the inhibition of T lymphocyte proliferation is Jagged-1, a Notch ligand [83]. Because Jagged-1 is expressed in MSCs suggests that Notch signaling is involved in MSC immunosuppressive functions. Liotta et al. showed that when Jagged-1 was blocked by antibodies, the MSCs inhibitory effects were decreased on a population of alloantigen-activated CD4^+^ lymphocytes [84].

## 4. The Modulation of MSC Immunoregulatory Properties

Many studies suggest that MSCs and immune cells have established two-way regulatory mechanisms; thus, the activation of MSC immunoregulatory properties requires the presence of derived proinflammatory cytokines from immune cells. Similarly, as a result of this activation, factors secreted by MSCs also regulate immune response.

### 4.1. MSC Activation Requires an Inflammatory Environment

MSCs must be “activated” to efficiently perform their immunoregulatory role [2]. Broad evidence demonstrates that this “activation” requires the presence of proinflammatory cytokines derived from T lymphocytes, macrophages, and NK cells, thus indicating that there are bidirectional regulatory mechanisms between MSCs and immune cells. Cytokines, such as IFN*γ*, are necessary in this process, either alone or in combination with TNF*α*, IL-1*α*, IL-1*β*, or IL-17 [14, 17, 85] (Figure 2). The importance of an inflammatory environment for MSC immunosuppressive capacity has been shown both* in vitro* and* in vivo.* The initial experiments indicated that when exposed to IFN*γ*, MSCs induce the expression of IDO [13, 17] and PD-L1 [34] and increase the secretion of PGE_2_ [14, 60]. Similarly, the conditioned medium of MSC- and IL-15-activated NK cell cocultures can inhibit NK-cell proliferation, which indicates that this coculture activates MSC immunoregulatory properties [27]. Other studies have shown that NK cells can produce IFN*γ* when they interact with MSCs (autologous or allogeneic), and MSC exposure to IFN*γ* increases the expression of HLA-I in MSCs, which reduces the secretion of cytokines and cytotoxic activity of NK cells [51] (Figure 2). This evidence suggests that bidirectional regulatory mechanisms drive the interaction between MSCs and NK cells. MSCs express NK receptor ligands, such as PVR, Nectin-2 (NAM-1 ligands), and ULPBs and MICA (NKG2D ligands). Because of these receptor-ligand profiles, it is likely that the interaction of NK cells and MSCs (autologous or allogeneic) results in IFN production by NK cells, whereas this exposure of MSCs to IFN*γ* increases HLA-1 expression in MSCs and other immunoregulatory molecules, thereby reducing the secretion of cytokines and NK cytotoxic activity [51]. In addition to the abovementioned cytokines, a recent study reported that IL-17 together with IFN*γ* and TNF*α* increased inhibition of T-cell proliferation mediated by MSCs, apparently through a synergic effect of the three cytokines, leading to a high expression of inducible nitric oxide synthase (iNOS) [85].

Ankrum et al. recently suggested that MSCs are not fully immunoprivileged because of their capacity to activate cells of the immune system (NK cells, macrophages, etc.) and may even be rejected by such immune cells [86]. Thus, resting MSCs are immunogenic and able to promote the secretion of inflammatory cytokines, which in turn induce MSC to express and secret distinct immunoregulatory molecules, allowing them to evade the immune response. In this regard, it has been demonstrated that naïve MSCs or primed previously with INF*γ* plus TNF*α* are able to decrease T-cells proliferation. However, naïve MSCs induce the secretion of INF*γ* and IL-2 by activated T cells at two days of coculture and this is a cellular event prior to inhibition of proliferation. The authors suggested that unprimed MSC transiently induce proinflammatory cytokines secretion, which promote the increase of their immunoregulatory capacity [87].

*In vivo* studies in mouse models suggest that MSCs are effective in the treatment, but not prevention of GVHD. The highest survival rates were obtained when MSCs were administered when serum IFN*γ* concentrations peaked. The injection of MSCs preactivated with high concentrations of IFN*γ* was effective in GVHD prevention and resulted in 100% survival. However, the injection of MSCs with low concentrations of IFN*γ* did not increase survival [88].

Similar results were observed in clinical trials. The administration of MSCs to patients with steroid refractory GVHD significantly improved patient outcomes [89, 90]. However, when MSCs were administered simultaneously with hematopoietic stem cells (HSC), there was no change in grade II/IV GVHD incidence and a high incidence of relapse [91].

### 4.2. MSCs and Toll-Like Receptors

Toll-like receptors (TLRs) are expressed by many immune cells, and their principal function is to detect pathogens. The activation of TLRs is essential to initiate an innate immune response and supports the adaptive immune response. Ten TLR types have been identified in humans and each recognize specific molecular patterns associated with bacterial, viral, or fungal pathogens. The signaling pathway common to all TLRs is the activation of NF-*κβ*, which controls the expression of several inflammatory cytokines and the expression of maturation markers [92]. Different TLRs are involved in autoimmune diseases, chronic inflammation, and infections [93].

MSCs express TLR-2, 3, 4, 5, 6, 7, and 9, which are all functional (except TLR-9) because its activation results in receptor internalization and the activation of the NF-*κβ*, MAPK, and AKT pathways [62, 84, 94]. The initial analyses of TLR participation in MSC-mediated immunoregulation described contradictory results. An* in vitro* population enriched with alloantigen-activated CD4^+^ lymphocytes cultured in the presence of poly(I:C) or LPS-treated MSCs, activated TLR-3 and TLR-4 in the MSCs, thus inhibiting their immunosuppressive capacity. This effect may be because of the reduced expression of Jagged-1 in MSCs [84]. Contradictory results by Opitz et al. showed an augmentation in immunosuppressive capacity after TLR-3 and TLR-4 activation in MSCs, which continued to inhibit T-cell proliferation, even in low MSC : T cells ratio in cocultures [94]. These contradictory results can be explained with additional evidence from Tomchuck et al. who observed that TLR-3 activation in MSCs supports the activation of the anti-inflammatory cytokines IL-10 and IL-12, whereas TLR-4 activation supports the secretion of proinflammatory cytokines [62]. These results were corroborated by Waterman et al. who reported that TLR-3 supported MSC immunosuppressive effects, whereas TLR-4 supported proinflammatory effects. These authors proposed that MSCs could be polarized to one of two phenotypes: proinflammatory or anti-inflammatory. Each MSC population may possess unique characteristics and differ in their secretion of cytokines, differentiation capacity, extracellular matrix deposits, TGF_*β*1_-signaling pathways, and expression of Jagged, IDO, and PGE_2_ [95]. However, the TLR participation in the immunoregulation by MSCs remains controversial. A recent study demonstrated the secretion of proinflammatory cytokines by MSCs upon activation of TLR-3 or TLR-4 [96].

## 5. Immunoregulation in Alternative Sources of BM-MSCs

Currently most studies on the biology of MSCs have used BM-derived MSCs. Similarly, most literature describing the molecules that participate in immunoregulatory mechanisms are specific to BM-MSCs [8, 13, 14, 16, 17, 20, 28, 31, 32, 38]. Few studies have used other sources such as PL [24, 76, 97–99], UCB [24, 60, 98], UC [34, 98, 100–102], AT [8, 99, 101–103], and WJ [8, 98, 99, 101, 104] (Table 1). Immune cell studies that have used MSCs derived from PL, AT, UCB, WJ, placental villi, amnion, and chorion have focused principally on T lymphocytes, one of the most studied cell types in MSC immunoregulation [8, 24, 76, 97, 101–103, 105–111] (Table 2). The search for alternative sources is particularly important not only because obtaining BM is expensive and invasive, but also because of reports that MSC differentiation capacity diminishes as the individual ages [112, 113].

Some reports have compared the immunoregulatory properties of MSCs from different sources to determine which is the most viable for BM replacement. In this regard, our research group has demonstrated that MSCs from BM and UCB, have identical immunoregulatory capacity [24]. However we have shown, in the same way as other groups, that in contrast to BM-MSCs, PL-derived MSCs have a lower capacity [24, 107]. In fact, results relating to PL are contradictory between groups [76, 97, 99, 108]. In this regard, results of a preclinical study for treatment of 9 patients with grades III-IV acute GVHD with PL-MSCs showed complete and partial recovery in two and four patients, respectively. No recovery was observed in two patients and one patient presented with convulsions [114]. The inconsistency of the results observed in clinical application of PL-MSCs could be related to the inconsistent results obtained* in vitro* by various groups. A different study comparing UC-, UCB-, PL-, and WJ-derived MSCs observed differences in the capacity to express HLA-G or TGF_*β*1_ after activation with IFN*γ*. There were no differences observed in IDO secretion [98]. Screening for immunosuppressive factors in WJ- and BM-MSCs activated with IFN*γ* or TNF*α* has indicated differences in the postactivation secretion of IDO, HGF, and PGE_2_, which may influence immunoregulatory properties [104]. It is important to pursue comparative studies to determine whether MSCs from alternative sources operate with the identical immunoregulatory mechanisms as BM-MSCs, which are used in cellular therapy. These studies will be vital in determining alternative sources of MSCs for their potential implementation at the clinical level.

## 6. MSCs and Clinical Applications

The use of stem cells to replace cells and tissues damaged by congenital or degenerative disease or trauma is called stem cell therapy. In such procedures, cells are administered to patients through the blood or directly to the damaged tissue. The use of stem cells in cellular therapy is a current topic of debate. We previously outlined that MSCs have three biological properties that make them potential candidates for this use: high differentiation potential, trophic factor secretion, and immunoregulatory capacity. MSCs are potentially applicable to many diseases, such as GVHD, autoimmune diseases and bone, cartilage, and cardiovascular diseases. The beneficial effects of MSCs administration regarding several of the aforementioned diseases have been analyzed in animal models and phases I, II, and III clinical studies have been initiated [115, 116]. In the following sections, we focus on the use of MSCs in the treatment of immune-associated diseases with special emphasis on the treatment of GVHD.

### 6.1. Graft versus Host Disease

Because of their immunoregulatory properties, BM-MSCs have been applied principally in HSC transplants because MSCs are capable of treating and preventing GVHD [89, 117–119]. After HSC transplant, GVHD presents when donor T lymphocytes recognize patient HLA molecules (alloantigen) as nonself and mount an immune response (allogeneic immune response). GVHD can be acute or chronic depending on the time of onset and intensity of tissue damage. Acute GVHD (aGVHD) appears within the first 100 days of the transplant, whereas chronic GVHD (cGVHD) has a later onset. Although the exact pathophysiology is unknown, three phases are believed to describe aGVHD onset: (1) the activation of host antigen presenting cells (APCs) by the transplant conditioning regimen (radiotherapy and/or chemotherapy); (2) the activation of T lymphocytes, which proliferate and differentiate in response to histoincompatible antigens presented by APCs; and (3) a cellular effector and inflammatory phase. The final phase is a combined effect of different sectors of the immune system (cytotoxic T lymphocytes and NK cells) and inflammatory cytokines (IFN*γ*, TNF*α*, IL-1, etc.), which together promote inflammation and tissue damage in various organs and can cause death [120].

The first clinical trial evaluating the safety and effectiveness of MSCs in the treatment of GVHD was performed by Frassoni et al. who observed that the coinfusion of HSC and nonirradiated MSCs from the identical donor reduced the incidence and severity of GVDH in recipients of an allograft from an HLA-identical sibling [121]. Subsequently, Le Blanc et al. reported the case of a 9-year-old boy with steroid refractory aGVHD grade IV who was treated with haploidentical MSCs from his mother. The child improved significantly on the 4th day after infusion. Approximately 70 days after treatment, the child had renewed symptoms of diarrhea and high levels of bilirubin. The administration of a second dose of MSCs significantly improved the patient's condition, and he remained stable throughout the following 100 days [121]. After this case, the identical group reported another study with the administration of MSCs to 8 steroids refractory GVHD grades III-IV patients. No toxic effects were observed, and survival was significantly improved compared to the control [89]. In a multicenter phase II study of 55 steroid refractory GVHD patients. A response was observed in 39 patients treated with MSCs, of which 30 recovered completely and 9 partially recovered. Survival was significantly improved in patients experiencing full recovery, and the mortality rate was significantly lower than that generally associated with the transplant. The authors suggest that the effect of the MSCs was independent of the donor because MSCs of HLA-identical, haploidentical, and incompatible siblings had similar results [90].

Other studies on the use of MSCs have reported less encouraging results. von Bonin et al. treated 13 patients with steroid-refractory aGVHD with different doses of MSCs (1–5 applications) from unrelated donors that had been expanded in medium with platelet lysate. Only 5 patients responded to treatment and only 4 were alive after 257 days of treatment [122]. Similarly, Müller et al. reported the treatment of 5 children with acute or cGVHD. MSCs doses were increased in accordance with cell availability from 0.4 × 10^6^ to 3.0 × 10^6^/kg. Only one patient responded to this treatment [123]. In addition, it has been suggested that the cotransplant of HSC and BM-MSCs may prevent GVHD, but there is a high rate of relapse [91]. However, a clinical trial with 37 children treated with multiple doses of BM-MSCs for steroid-refractory grades III-IV aGVHD reported complete response in 24 children and partial response in 8 children [124]. The contradictory results may be because of differences in the method of MSC expansion, the number of cells administered, number of doses given, diagnosis of the recipient (chronic or aGVHD), stage of GVHD development, and administration of MSCs, among others. In attempts to eliminate some these variables, clinical studies have been performed with a universal BM-MSC preparation, such as Prochymal, which has rendered positive results in the treatment of GVHD [125]. Additionally, the effects of using alternative sources of MSCs, such as fetal membranes [114] or UC [126], have been studied.

Some studies have analyzed systemic changes after administration of MSCs. Jitschin et al. reported that, in steroid-refractory GVHD patients after MSC infusion, a high frequency of CD4^+^CD25^med-hi^CD127^lo^Foxp3^+^ and Tr1 populations was detected. A decrease of Th17 cells and no changes in the number of NK or B cells were observed. Furthermore, the authors suggest an induction and maintenance of Tregs, because high levels of IL-2 were detected [42]. In another similar study an increment of CD8^+^CD28^−^ and CD5^+^CD19^+^ populations and decrease of CD8^+^CD28^+^ and CD5^−^CD19^+^ B cells after BM-MSC infusion in the responsive group were reported [127]. The first two populations of T and B cells are related with the maintaining of peripheral tolerance or the induction of differentiation of Tregs [127]. Similar results were obtained recently in 23 refractory cGVHD patients treated with MSCs in which it was detected an increment in the population of CD19^+^CD5^+^IL-10^+^ B cells and a high plasma concentration of IL-10 [128]. It is important to mention that it has been suggested that changes in the immune microenviroment in patients may promote the risk of infections. In this regard, a retrospective cohort study showed that treatment of GVHD with MSC is a risk factor for pneumonia related death [129]. Furthermore, Remberger and Ringdén reported treatment with MSC increment incidence of invasive fungal infections [130]. Finally, randomized Phase III trials are necessary to determine the effectiveness of MSCs in GVHD.

### 6.2. Autoimmune Diseases

MSC immunosuppressive capacity may be useful in the treatment of autoimmune diseases. In these pathologies, self-antigens are not tolerated, and the body mounts an immune response against its own tissues and organs. The resulting damage can be systemic (systemic lupus erythematosus) or targeted to a specific organ or tissue, such as the pancreas (type I diabetes), central nervous system (multiple sclerosis), or joints (rheumatoid arthritis) [131–133]. Animal models have demonstrated that MSC treatment is effective in some of these conditions, several of which have also been clinical trials, as described below [132, 133].

Systemic lupus erythematosus (SLE) is a chronic autoimmune disease that affects connective tissue. Clinical studies using allogeneic BM-MSCs [134] and UC-MSCs [135, 136] to treat this disease have described positive outcomes and no severe side effect. In a multicenter study, 40 SLE patients were intravenously transplanted with UC-MSCs and total or partial clinical response was observed in 60% of patients. The beneficial effect of UC-MSC administration is not permanent, because 29.2% of patients relapsed [137]. Although these results are interesting, it is important to mention that in the future a controlled and randomized study will be necessary to conclusively demonstrate the effectiveness of UCB-MSC administration for SLE treatment [137]. Although the mechanism through which MSC improves patient condition is unknown, Li et al. reported increase of CD4^+^CD25^+^Foxp3^+^ Tregs in peripheral blood of refractory SLE patients transplanted with UC-MSCs, even after one month of treatment. Additionally, a significant decrease of Th17 cells since the first week and up to twelve months after transplant was observed [138]. Also, Wang et al. detected an increase in IDO activity after administration of UC-MSCs in SLE patients, evidenced by kynurenine concentrations [135]. These results suggest participation of immunoregulatory mechanism regarding beneficial effects of MSC administration in such patients.

On the other hand, some reports have shown that BM-MSC administration in murine model of SLE do not decrease the levels of autoantibodies or the mortality rates [139]. Furthermore, using the same animal model, Youd et al. reported that administration of allogeneic BM-MSCs to prevent or treat SLE enhances autoantibody production and even exacerbates the disease in both experimental conditions [140]. Also, a study carried out in two patients showed that administration of autologous BM-MSCs increased peripheral blood CD4^+^CD25^+^Foxp3^+^ Tregs cells, but no improvement was observed in patients [43]. Today, there are no clinical trials in progress to assess efficacy of MSC transplantation in the treatment of Lupus erythematosus [http://www.clinicaltrials.gov/].

Type I diabetes is a chronic metabolic disease characterized by an autoimmune reaction against insulin-producing pancreatic *β* cells.* In vitro* studies using cocktail of growth factors or genetic manipulation have shown that BM-MSCs can differentiate into insulin-producing cells [141]. However, such transdifferentiation capacity of BM-MSCs into endocrine pancreas cells in animal models has yielded contradictory results and still remains elusive. Nevertheless, in animal models have been demonstrated that MSCs can revert type I diabetes, enhances insulin secretion and sustain normoglycemia [131]. It has been proposed that such beneficial effects are mainly due to trophic factors and immunoregulators secreted by MSCs and not to their differentiation capacity [141].

In a clinical trial, the administration of insulin-producing cells derived of MSCs from adipose tissue in 11 patients reduced insulin requirements over the first 2 to 4 months after intraportal infusion [142]. In another trail, WJ-MSCs were administrated to 15 patients and a decrease in insulin requirements and blood glucose levels was observed even after 24 months of follow-up [143]. The exact mechanism of this therapeutic effect is unknown. However, it is thought that MSC immunoregulatory capacity prevents the destruction of the *β*-pancreatic cells rather than their differentiation capacity to regenerate *β* cells. In that regard, intravenous administration of BM-MSCs in a murine model of type 1 diabetes reversed hyperglycemia and improved pancreatic regeneration, which is associated with an increase of CD4^+^CD25^+^Foxp3^+^ Tregs cells in spleen and pancreatic lymph nodes [144]. Similar results were obtained by intraperitoneal administration of MSCs from AT-MSCs in mouse models, during early development of induced type I diabetes. Increased secretion of insulin and decreased glucose levels in peripheral blood were observed but also inflammatory infiltrate in pancreatic islets. Furthermore, a low frequency of CD4^+^IFN*γ* ^+^ and CD4^+^TNF*α* ^+^ T-cells was observed in pancreatic lymph nodes (PLNs), and in contrast, a high frequency of CD4^+^CD25^+^Foxp3^+^ Tregs, a decrease of IFN*γ* concentration and increase in TGF_*β*1_ were observed in pancreatic tissue [145].

Additionally, cotransplantation with BM-MSCs improves engraftment of pancreatic islets in humanized diabetic mouse model. Normoglycemia was maintained during 4 weeks after transplant which show that MSCs promote functionality of transplanted islets. The authors observed low infiltration by CD3^+^ T cells in the transplanted tissue and increase of CD4^+^CD25^+^Foxp3^+^ Tregs in peripheral blood [146]. Future clinical trials will be required to determine if such immunological mechanisms also occur in humans. Currently, phases I, II, and III clinical trials are evaluating the efficacy of autologous BM-MSCs [clinicaltrials.gov identifier: NCT02057211, NCT01068951, NCT01157403] and UC-MSCs in the treatment of type I diabetes [clinicaltrials.gov identifier: NCT01374854].

Two autoimmune diseases affect the central nervous system (CNS): multiple sclerosis (MS) and amyotrophic lateral sclerosis (ALS). MS is a chronic inflammatory disease characterized by the loss of myelin and axon damage. ALS selectively targets motor neurons in the brain and spinal cord. Several studies have demonstrated in murine models that MSC has positive effects for prevention and treatment of both pathologies, but it is not known what mechanisms are involved in such process. Although* in vitro* studies show that, in the appropriate culture medium, MSCs differentiate into cell types with neuronal and glial characteristics, contribution of such mechanisms for* in vivo* tissue regeneration is controversial. In contrast,* in vitro* and* in vivo* studies have suggested that immunoregulation and trophic factor secretion are the main mechanisms used by MSCs to improve the symptoms of MS and ALS [1, 147]. Studies in a murine model with experimental autoimmune encephalomyelitis (EAE) as a model for multiple sclerosis have demonstrated that the intravenous administration of BM-MSCs improved the symptoms of the disease by decreasing central nervous system inflammation and demyelination. Other studies have reported that, in EAE mice that had received MSCs, less infiltration by CD3^+^ T cells and macrophages in CSN was observed after pathological analysis [148]. Also, it has been detected low levels of IL-17 and TNF*α* in serum [149]. Morando et al. observed that intravenous or intrathecal administration of BM-MSC promotes generation of CD4^+^Foxp3^+^ in CNS and also an increase of IL-17 mRNA [147]. Furthermore, after administration of hUC-MSCs in a murine EAE model, the improvement of disease activity is accompanied by an increase of CD4^+^CD25^+^Foxp3^+^ Tregs and decrease of Th17 in spleen, while in the spinal cord increased IL-4 and IL-10 and decreased IL-1 and IL-6 levels were observed [150]. Taken together, these results suggest that immunoregulation by MSCs has a neuroprotector effect that reduces demyelization and axonal loss and therefore results in the improvement of EAE symptoms. It is important to mention that in a recent study it was reported that BM-MSCs transplanted in a murine EAE model, CD8^+^ T cell infiltrate was increased in CNS, which exacerbated EAE [151]. The difference between these results could be due to disease mechanisms that underlie various models of EAE.

Similarly, allogeneic MSCs administration in murine models of experimental ALS showed an improvement in survival and motor function, in the spinal cord from MSC-treated mice [152]. Intrathecal infusion of BM-MSCs in a murine model of ALS delayed disease progression and prolonged survival [153]. Activated microglia secrete inflammatory molecules including TNF*α* and nitric oxide that play an important role in ALS and administration of hMSCs decrease microglial activation (CD11b^+^ cells) and concentration of TNF*α* in the spinal cord [153]. Based on the positive results obtained in murine models, clinical trials have been carried out to determine safety and efficacy of MSCs for the treatment of these pathologies. A clinical trial in which 34 MS and ALS patients received intrathecal or intravenous autologous MSCs reported that MSC administration was safe and had an immediate immunosuppressive effect that diminished inflammation [154]. A study by Mazzini et al. studied the direct application of autologous MSCs to the spinal cord in ALS patients and reported that MSC administration generated no adverse side effects and was safe; however there was no significant improvement in patients [155]. Currently, no study has shown an improvement in patient's condition by MSC treatment; therefore, further clinical trials must be performed. Several of such studies, analyzing the efficacy and safety of autologous or allogeneic MSCs for the treatment of MS and ALS, are in progress [http://www.clinicaltrials.gov/].

Rheumatoid arthritis (RA) is a chronic, systemic inflammatory disorder that primarily affects joints and results in bone and cartilage destruction. Collagen-induced arthritis (CIA) initiated in susceptible strains of mice by immunization with native type II collagen serves as a model of human rheumatoid arthritis. Several authors have used this animal model to analyze MSC efficacy to improve the symptoms of this disease; however evidence remains equivocal as conflicting results have been reported. Results in this animal model suggest that inflammatory microenvironment present in RA could reverse the immunosuppressive capacity of MSCs. Djouad et al. [156] observed that MSC administration does not improve the course of disease and* in vitro* experiments showed that TNF*α* was responsible of reverse biological function of MSCs. Additionally, it has been reported that the intravenous administration of MSCs has no effect on the progression of the disease [157] or make RA worse [158]. Similar results were reported by Papadopoulou et al. who observed immunoregulatory capacity of MSCs* in vitro*, but* in vivo*, they lost this capacity when they are administrated in the inflammatory RA environment [159]. In a recent study similar immunosuppressive capacity by synovium-derived mesenchymal stem cells (S-MSC) from AR patients and those from healthy donors was demonstrated. Both MSCs are capable of decrease proliferation of PBMC activated with PHA or alloantigens from healthy donors and also of autologous synovial T cells activated with PHA. However, when cocultures are added with exogenous IL-17 and/or TNF*α*, S-MSCs from AR patients or healthy donors, they lost their immunoregulatory capacity [160]. In addition, it has been reported that infusion of allogeneic-related HLA matched or partially matched MSCs does not affect RA development, while MHC mismatched MSCs exacerbate the disease activity [161].

In contrast to these results, other studies have shown that administration of syngeneic, allogeneic, or xenogeneic MSCs improves RA in mice models. Thus, González et al. showed that intraperitoneal administration of human AT-MSCs in mice with CIA reduced the incidence and severity of disease. Improvement of RA is accompanied by a decrease both in inflammation and proinflammatory cytokine secretion (IL-1*β*, IL-12, IL-17, TNF*α*, IFN*γ*, etc.) and reduction in Th1 and Th17 numbers. In contrast, expansion of CD4^+^CD25^+^Foxp3^+^ Tregs and IL-10 secretion was increased [162]. Similar results have been shown with human gingiva-derived mesenchymal stem cells [163]. In addition, in a RA murine model induced by antigens, intra-articular infusion of BM-MSCs prevented cartilage damage reduced the inflammation and also decreased serum concentration of TNF*α* [164].

A few studies have been done to determine safety and efficacy of MSCs administration to humans for the treatment of RA. A study carried out in four patients with refractory RA and treated with allogeneic MSCs from BM (1 patient) or UC (3 patient) administered intravenously showed no adverse effects; however clinical remissions were not detected [165]. In contrast, a clinical assay with 172 patients with active RA showed that UC-MSC administration is safe and that the improvement in patients is accompanied by an increase of CD4^+^CD25^+^Foxp3^+^ Tregs in peripheral blood and a decrease of TNF*α* secretion [166]. The same authors suggest the need for large multicentre trials. In this regard, two phases I and II clinical trials are currently performed to evaluate the efficacy of UC-MSCs [clinicaltrials.gov identifier: NCT01547091 and NCT01985464].

## 7. Conclusion

BM-derived MSCs have an immunoregulatory capacity because they can regulate the function of multiple immune system components. To fulfill this role, MSCs must be activated by proinflammatory cytokines such as IFN*γ*. MSCs can inhibit DCs maturation and thus prevent the activation of T lymphocytes and even more and decrease the proliferation and cytotoxic activity of NK cells. As a result of these characteristics, MSCs are a promising alternative treatment for immune-related diseases. Currently, MSCs have been used in the treatment of autoimmune diseases, including GVHD, and have rendered positive results. Despite this encouraging debut in clinical application, it is necessary to perform more clinical trials that extend the current knowledge of the biology of MSC immunoregulatory activity to optimize and control the patient's immune response for maximum benefits. These studies will be relevant to clinical decisions in the treatment of GVHD, autoimmune diseases, and other illnesses with an immune component, such as cancer, in which MSCs play an important role in the tumor microenvironment that favors growth as our group has previously demonstrated [10].

## Figures and Tables

**Table 1:** Description of the molecules involved in immunoregulation mechanisms by MSCs from different sources.

Sources of MSC	Molecules involved in MSC immunosuppression	Reference
Bone marrow	IDO, TGF_*β*1_, HGF, IL-10, HLA-G, PDL-1, PGE_2_	[8, 13, 14, 16, 17, 20, 28, 31, 32, 34, 38, 45, 46]

Placenta	IDO, TGF_*β*1_, IL-10, HLA-G, PDL-1	[24, 67, 76, 97, 98]

Umbilical cord blood	IDO, TGF_*β*1_, HGF, HLA-G, PDL-1, PGE_2_	[24, 60, 98]

Umbilical cord	IDO, TGF_*β*1_, HGF, IL-10, HLA-G, PDL-1, PGE_2_	[34, 98, 100, 101]

Adipose tissue	IDO, TGF_*β*1_, HGF, IL-10, PGE_2_	[8, 101]

Wharton's jelly	IDO, TGF_*β*1_, HGF, IL-10, HLA-G, PGE_2_	[8, 98, 101, 104]

**Table 2:** Comparison of *in vitro* immunoregulatory effects of human MSCs from different sources on T cells.

Sources of MSC	Source of T cells and activation	Immunoregulation	Reference
Bone marrow placenta	PBMC or mononuclear cells from umbilical cord blood activated with PHA or alloantigens	Similar immunoregulatory capacity	[105]

Bone marrow placenta	PBMC activated with PHA or alloantigens	MSCs from placenta have a higher immunoregulatory capacity than those from bone marrow	[76, 97]

Bone marrow adipose tissue	PBMC activated with alloantigens	Similar immunoregulatory capacity	[106]

Bone marrow adipose tissue umbilical cord blood Wharton's jelly	PBMC activated with PHA	Similar immunoregulatory capacity	[101]

Bone marrow umbilical cord	T cells activated with alloantigens	Similar immunoregulatory capacity	[34]

Bone marrow Wharton's jelly	PBMC activated with PHA PBMC activated with alloantigen	Similar immunoregulatory capacity WJ-MSCs are more potent in suppressing PBMC proliferation than BM	[104]

Bone marrow placenta	CD3^+^ or CD4^+^ T cells activated with PHA or anti-CD3/CD28	MSCs from bone marrow have a higher immunoregulatory capacity than those from the placenta	[107]

Bone marrow adipose tissue Wharton's jelly	CD3^+^ T cells activated with PHA or alloantigens	Similar immunoregulatory capacity	[8]

Bone marrow term fetal membrane umbilical cord placental villi	PBMC activated with alloantigens CD3^+^ T cells activated with anti-CD3/CD28	MSC from fetal membranes and umbilical cord are immunoregulators. Inconsistent results are observed in MSC from placenta	[108]

Placenta bone marrow adipose tissue	Mononuclear cells from umbilical cord blood activated with anti-CD3/CD28	Similar immunoregulatory capacity	[109]

Bone marrow umbilical cord Wharton's jelly placenta amnion	PBMC activated with alloantigens	Five sources have similar immunoregulatory capacity	[110]

Bone marrow adipose tissue	PBMC proliferation activated with anti-CD3/CD28	AT-MSCs are more potent in suppressing PBMC proliferation	[111]

Bone marrow adipose tissue	PBMC activated with anti-CD3/CD28	Similar immunoregulatory capacity	[103]

Bone marrow adipose tissue umbilical cord matrix	PBMC activated with PHA	MSCs from adipose tissue have a higher immunoregulatory capacity than those from the UCB or BM	[102]

Bone marrow umbilical cord blood placenta	CD3^+^ T cells activated with anti-CD3/CD28	MSCs from placenta have less immunoregulatory capacity than those from umbilical cord blood or bone marrow	[24]

Bone marrow adipose tissue Wharton's jelly placenta	T cells activated with PHA/IL-2	MSCs from three sources have a higher immunoregulatory capacity than those from BM. WJ-MSCs have the best immunoregulatory capacity	[99]
